# The Hippocratic Oath

**Published:** 1897-08

**Authors:** 


### The Hippocratic Oath.

A correspondent of the N. Y. *Medical Record* seeks information regarding the Hippocratic oath, taken by physicians upon graduation.

He states that he has inquired as to the substance of this oath of many physicians, who have been unable to give him a satisfactory answer. It is highly probable that but a few of our best educated physicians ever knew the text of the oath they were taking. The *Medical Record* gives the following translation of the oath in full:

"I swear by Apollo the physician, and Æsculapius, and Health, and All-heal, and all the gods and goddesses, that, according to my ability and judgement, I will keep this oath and this stipulation—to reckon him who taught me this Art equally dear to me as my parents, to share my substance with him, and relieve his necessities if required; to look upon his offspring on the same footing as my own brothers, to teach them this art, if they should wish to learn it, without fee or stipulation; and by precept, lecture, and every mode of instruction, I will impart the knowledge of the Art to my sons, and those of my teachers, and to disciples bound by stipulation and oath according to the law of medicine, but to none others. I will follow that system of regimen, according to my ability and judgment, I consider for the benefit of my patients, and abstain from whatever is deleterious and mischievous. I will give no deadly medicine to any one if asked, nor suggest any such counsel; and in like manner I will not give to a woman a pessary to produce abortion. With purity and with holiness I will pass my life and practice my Art. I will not cut persons laboring under the stone, but will leave this to be done by men who are practitioners of this work. Into whatever houses I enter, I will go into them for the benefit of the sick, and will abstain from every voluntary act of mischief and corruption; and, further, from the seduction of females or males, of freemen and slaves. Whatever in connection with my professional practice or not in connection with I see or hear, in the life of men, which ought not to be spoken of abroad, I will not divulge, as reckoning that all such should be kept secret. While I continue to keep this Oath unviolated, may it be granted to me to enjoy life and the practice of the Art, respected by all men,. in all times. But, should I trespass and violate this Oath, may the reverse be my lot."

